# The Roles of Innate Lymphoid Cells in the Gastric Mucosal Immunology and Oncogenesis of Gastric Cancer

**DOI:** 10.3390/ijms24076652

**Published:** 2023-04-02

**Authors:** Yuhao Jiao, Zhiyu Yan, Aiming Yang

**Affiliations:** 1 Department of Gastroenterology, Peking Union Medical College Hospital, Chinese Academy of Medical Sciences & Peking Union Medical College, Beijing 100730, China; jiaoyuhao@pumch.cn (Y.J.); nirvanay@student.pumc.edu.cn (Z.Y.); 2 4 + 4 M.D. Program, Chinese Academy of Medical Sciences & Peking Union Medical College, Beijing 100730, China

**Keywords:** innate lymphoid cells, natural killer cells, mucosal immunology, *Helicobacter pylori*, gastric cancer

## Abstract

Innate lymphoid cells (ILCs) are a group of innate immune cells that have garnered considerable attention due to their critical roles in regulating immunity and tissue homeostasis. They are particularly abundant in the gastrointestinal tract, where they have been shown to interact with commensal bacteria, pathogens, and other components of the local microenvironment to influence host immune responses to infection and oncogenesis. Their tissue-residency properties enable gastric ILCs a localized and rapid response to alert and stress, which indicates their key potential in regulating immunosurveillance. In this review, we discuss the current understanding of the role of ILCs in the gastric mucosa, with a focus on their interactions with the gastric microbiota and *Helicobacter pylori* and their contributions to tissue homeostasis and inflammation. We also highlight recent findings on the involvement of ILCs in the pathogenesis of gastric cancer and the implications of targeting ILCs as a therapeutic approach. Overall, this review provides an overview of the diverse functions of ILCs in gastric mucosa and highlights their potential as targets for future therapies for gastric cancer.

## 1. Introduction

Innate lymphoid cells (ILCs) have emerged as a fascinating group of immune cells that share a common lymphoid progenitor origin with T and B lymphocytes [1]. However, what sets ILCs apart from these canonical lymphocytes is their lack of rearranged antigen receptors, highlighting their innate immunity nature [1]. As a crucial component of the innate arm of the human immune system, ILCs have gained growing attention due to their distinctive abilities in maintaining tissue homeostasis and mucosal immune responses [1]. In the past half-century, new members of the ILC family have been gradually identified following the discovery of natural killer (NK) cells, which were initially found to exert a cytotoxic effect against virus infection [1,2]. Lymphoid tissue inducer (LTi) cells were then identified to have a critical function in inducing the formation of lymph nodes in embryogenesis [3]. The development of these two prototypes of ILCs was initially found to rely on the downstream signaling transduction via common γ chain (γc; i.e., interleukin-2 receptor γ), IL-7 receptor α (IL-7Rα), and transcription factor DNA binding 2 (ID2) [3,4,5,6]. In the past twenty years, multiple distinct cell subsets that share developmental dependence of γc, IL-7Rα, and ID2 were identified and are now classified into the superfamily of ILCs. Intriguingly, some ILC subsets were found to have similar transcriptional features and immune response patterns to T helper (Th) cells [1]. For example, a subset of the retinoic acid receptor-related orphan receptor-γ t (RORγt) expressing cells, like Th17 cells, could exert the Th17 axis immune response by secreting IL-17 and IL-22 in response to IL-23 and IL-1β [7]. However, what distinguishes ILCs from T and B lymphocytes the most is the lack of recombinant activating gene (RAG) expression [8], resulting in the absence of a diverse and specific antigen-receptor repertoire [1].

ILCs are typically divided into three main groups based on their signature cytokine production and reliance on specific transcription factors during development [9]. Group 1 ILCs are dependent on the T-box transcription factor, TBX21, also known as T-bet, and include both the cytotoxic NK cell population and the helper-like ILC1 subset (ILC1s), which both secrete interferon-γ (IFN-γ) when activated [10,11,12]. Group 2 ILCs (ILC2s) are characterized by their dependence on the transcription factor GATA-3 and their secretion of Th2-associated cytokines, such as IL-5 and IL-13 [13,14]. Group 3 ILCs (ILC3s) are RORγt-dependent and secrete signature cytokines, IL-17 and IL-22 [7]. This group consists of several subsets, including the C-C Motif Chemokine Receptor 6 (CCR6)-expressing lymphoid tissue inducer (LTi) cells, as well as both natural cytotoxicity receptor (NCR)+ and NCR- ILC3s [7,9]. What distinguishes ILCs from other innate immune cells and adaptive immune cells are their tissue-residency properties of ILCs and rapid response to stimuli, enabling them as a key player in maintaining mucosal homeostasis, especially in the gastrointestinal mucosa [8]. Their ability to communicate with other immune subsets and direct different immune responses also makes them an essential target for studying the pathogenesis of various diseases. Abnormalities in ILC activity have been implicated in the pathogenesis of inflammatory bowel diseases, gastroenteric infections, and cancers, which highlights the importance of understanding ILC immunology [15,16,17].

Gastric cancer is a major worldwide health problem, particularly in East Asian countries [18]. In 2020, over 1 million diagnosed cases of gastric cancer resulted in approximately 770 thousand deaths, making it the third leading cause of cancer-related deaths [19,20]. The current first-line treatment for gastric cancer involves surgical or endoscopic resection of resectable tumors and systemic chemotherapies for unresectable advanced or metastatic disease [20]. Immunotherapies, such as immune checkpoint blockade, have been approved to treat advanced disease [20,21]. However, the overall survival rate of advanced gastric cancer patients after administering immunotherapies and other targeted therapies remains unsatisfying, with a poor rate of only 12–15 months [22]. Thus, a better understanding of the biology of gastric cancer and the local immune microenvironment is crucial for the development of novel therapeutic strategies. In this review, we aim to summarize the mechanisms by which ILCs function in the gastric mucosa, mucosal immunity, and gastric microbiota. We will focus on the relationship between ILC abnormalities and the oncogenesis of gastric cancer, as well as their implications for treating such diseases.

## 2. ILCs in Maintaining Gastric Mucosal Homeostasis and Regulation of Mucosal Immunity

The key machinery that allows ILCs to respond to immunological stimuli is by sensing signals from the immune niche via their comprehensive activating, inhibitory, and cytokine receptors [23]. Unlike T or B lymphocytes, which mainly circulate in the periphery and lymphoid tissues as sentries to alert, the majority of ILCs express tissue-homing receptors and are tissue-resident, meaning that they have self-maintenance properties in situ without the need for continuous replenishment from circulating precursors [24]. Their tissue-residency properties allow for a prolonged, rapid, and localized immune response in epithelial tissues, such as skin, respiratory tract, gastrointestinal tract, urogenital tract, and salivary glands [25]. Without the necessity of triggering and initiating adaptive immune pathways via the prerequisite activation of antigen-presenting cells, ILCs are among the earliest responders to exogenous stimuli [26]. The rapid and localized immune response carried out by ILCs is of great importance in maintaining the homeostasis of mucosal immune reactions and local microorganisms. Upon exposure to pathogens and other stimuli, ILCs can secrete their unique spectrum of cytokines and communicate with other immune subsets, eventually directing different axes of immune responses. These features of ILCs make them critical in maintaining mucosal homeostasis and regulating mucosal immunity, including gastric mucosa.

NK cells, as the only cytotoxic subset of ILCs, have unique strategies to distinguish between self and non-self [27]. The pool of NK cells can be divided into two compartments, one that circulates in the blood and primary lymphoid organs and the other that resides in tissues such as the gastrointestinal intraepithelial layer and lamina propria layer [28,29]. Both compartments share similar biological features, including their ability to detect tissue damage signals, pathogen, virus-infected cells, and cancer cells through their multiple activating receptors, inhibitory receptors, and cytokine receptors [29,30,31]. The overall input of signals alters specific transcription factors and pro-apoptotic molecules, ultimately determining their activation, proliferation, or apoptosis [32]. During development, NK cells dynamically tune the threshold for activation by adjusting the expression level of their activating and inhibitory receptors in response to their crosstalk with microbes and the local microenvironment, which ensures an appropriate intensity of the immune response and limits autoimmunity [33,34]. A unique feature of gut mucosal NK cells is that their gain of normal functions depends on the priming by local commensal bacteria through dendritic cells [35,36]. Additionally, lack of exposure to microbes has been shown to significantly limit the NK cell function, demonstrated in a germ-free mouse model [37]. In turn, in gastric mucosal inflammation and the microenvironment of gastric cancer, adequately primed and activated NK cells mainly perform their anti-pathogen and anti-tumor effect via direct cytotoxicity and augment inflammation via their potent cytokine and chemokine-production capacity. Activated NK cells perform their direct cytotoxicity by releasing the pore-forming cytolytic molecule, perforin and granzymes, as well as using tumor necrosis factor (TNF)-related apoptosis-inducing ligand (TRAIL) pathways and antibody-dependent cellular cytotoxicity (ADCC) strategies [32]. Cytokines produced by NK cells, including TNF, IFN-γ, and granulocyte-macrophage colony-stimulating factor (GM-CSF), have a robust pro-inflammatory effect that recruits tissue-homing leukocytes and provokes downstream inflammation reactions [38]. Meanwhile, tumors escaping from NK cell-mediated immunosurveillance may also be achieved by inducing the expression of inhibitory receptors such as NKG2A on NK cells [39], as well as promoting the transdifferentiation from NK cells to other intermediate ILC1 subsets with reduced anti-tumor function in the presence of elevated levels of transforming growth factor-β (TGF-β) [40].

ILC1s are the helper-like ILC compartment of Group 1 ILCs that have no or limited direct cytotoxicity effect toward virus-infected cells or cancer cells but have substantial potential for cytokine and chemokine-production abilities, which is the primary reason that distinguishes them from NK cells [41,42]. Upon activation by IL-15, ILC1s are capable of producing IFN-γ and granzymes, which play a role in clearing infections and immunosurveillance of oncogenesis [11]. However, the tissue-specific mechanisms of anti-tumor responses by ILC1s in different tissues or cancer types remain a subject of debate owing to their comprehensive dynamics and plasticity [43,44]. In a liver cancer model, ILC1s control the metastatic seeding of tumor cells, whereas NK cells are more essential in limiting tumor growth itself [45]. It is gradually recognized that the borderline between the cytotoxic NK cells and non-NK helper-like ILC1s may not be strictly defined. The plasticity and transdifferentiation of Group 1 ILCs are closely related to TGF-β, which potentially alters the phenotype of Group 1 ILCs and may dampen their anti-tumor effect [40].

Regarding all the helper-like ILCs, studies on their distribution in murine and human gastric mucosal have shown that ILCs are dominated by ILC2s, with lesser Group 1 ILCs and limited amounts of ILC3s [46,47]. Therefore, ILC2s have received the most extensive research attention in gastric mucosal and gastric cancer immunology among all the helper-like ILC subsets. The regular roles of ILC2s in mucosal immunity are their contributions to maintaining mucosal integrity and tissue remodeling, as well as the anti-parasite effect [48]. In response to epithelial-derived cytokines, IL-33, IL-25, and thymic stromal lymphopoietin (TSLP), ILC2s produce type 2 cytokines, including IL-5, IL-9, and IL-13 [49,50]. The type 2 cytokines produced by ILC2 are pivotal in the downstream recruitment and activation of eosinophils, mast cells, and macrophages, as well as tissue repair and fibrosis [51,52,53]. The roles of ILC2s in tumor immunology have been studied in different tumor models with controversial implications. Their anti-tumor mechanism has been shown in pancreatic cancer and melanoma models that infiltrating and activating ILC2s by IL-33 could further recruit and activate dendritic cells and T cells [54]. Conversely, the impropriate production of IL-13 by ILC2 drives the differentiation and infiltration of myeloid-derived suppressor cells (MDSCs), which favor the growth and metastasis of multiple types of tumors, including prostate cancer, bladder cancer, and leukemia [55,56,57]. These findings indicate that ILC2s may have both pro-tumor and anti-tumor effects and further research is needed to clarify their precise role in tumor immunology. 

The actual contribution of ILC2s in the setting of gastric cancer is discussed in the following sections.

ILC3s are the key players in the intestinal (but not gastric) mucosa due to their high frequencies and close relationship with gut microbiota. Primed by commensal bacteria, ILC3s produce IL-22 and IL-33, which drives the production of antimicrobial peptides such as regenerating islet-derived (REG) IIIβ and REG IIIγ, fucosylation of epithelial cells to enhance mucosal integrity, and, in turn, activation of ILC2 via IL-33 [58,59]. The major histocompatibility complex (MHC)-expressing ILC3s presents antigens to CD4+ T cells, which triggers downstream adaptive immune responses in the setting of oncogenesis and favors the anti-tumor effect [17,60,61]. However, the plasticity of ILC3s, with the potential for transdifferentiation to regulatory ILCs or ILC1s induced by TGF-β, may limit their pro-inflammatory capabilities and diminish their anti-tumor responses [62]. In both physiological and inflamed gastric mucosa, ILC3s are present at low frequencies and may have limited contribution to local immune response.

In general, mucosal ILCs have critical roles in the maintenance of mucosal homeostasis, pathogen defense, and immunosurveillance of tumors. The close interaction between ILCs and local bacteria, both commensal and pathogenic, strongly influences ILC function and can alter the immune response to inflammation and oncogenesis. In the following sections, we will discuss the implications of crosstalk between ILCs and gastric bacteria for oncogenesis, as well as the direct contributions of ILCs to the immunosurveillance of gastric cancer.

## 3. Crosstalk between ILCs and Gastric Microbiota and Its Impact on Oncogenesis

Gastrointestinal mucosa is not sterile and constantly exposed to a vast number of microorganisms. ILCs are critical in maintaining the integrity of the mucosal barrier by acting as the first-line defenders against pathogenic bacteria such as *Helicobacter pylori* (*H. pylori*), which is now widely accepted as a significant cause of gastric inflammation and oncogenesis [63]. Secretion of cytokines such as IFN-γ by group 1 ILCs and NCR-ILC3s could contribute to the clearance of intracellular bacteria via promoting activation and phagocytosis by macrophages and dendritic cells [16]. Meanwhile, the IL-22 production by group 3 ILCs could further induce the production of multiple anti-microbial peptides, such as defensins and cathelicidins from epithelial cells [25,64], as well as RegIIIγ from both epithelial cells and ILC3s [65,66,67]. In addition, ILCs are also proposed to be directly involved in regulating gut epithelial integrity [16]. The epidermal growth factor amphiregulin and IL-13 derived from ILC2 could favor epithelial restoration by regulating enterocytes, goblet cells, and crypt stem cells [53,68]. IL-22 and lymphotoxin produced by ILC3s could induce the differentiation and proliferation of epithelial progenitor cells for local replenishment. Mucus production by goblet cells and fucosylation of enterocytes to enhance the gut barrier may also be promoted by ILC3s [69]. However, the roles of ILC1s in regulating epithelial integrity might be controversial. TGF-β produced by ILC1s could possibly contribute to epithelial stem cell proliferation and differentiation by inducing the expression of variant 6 of CD44 (Cd44v6) [70]. On the other hand, the pro-inflammatory cytokine IFN-γ secreted by ILC1s was demonstrated to dampen the epithelial barrier and worsen the disease severity in a celiac disease model [71]. In all, the maintenance of the gastrointestinal barrier and the clearance of pathogenic bacteria is of great importance in limiting local inflammation and potentially reducing the risk of oncogenesis.

Most studies on the roles of ILCs in gastrointestinal mucosal immunology and their crosstalk with local microorganisms are focused on the intestinal tract as opposed to the gastric mucosa. Gastric mucosa was, for a long time, believed to be sterile owing to the harsh acidic environment. Following the discovery of *H. pylori* and its close relationship with gastritis, gastric or peptic ulceration, and gastric cancer, a diverse community of commensal bacteria has been identified in gastric mucosa [72]. Collecting all these microorganisms residing on gastric mucosa is defined as gastric microbiota, gradually gaining attention due to their impact on local immune responses and the pathogenesis of multiple diseases.

Compared to the gut microbiota, which is estimated to consist of approximately 10^14^~10^15^ microorganisms with over 500 different species, the gastric microbiota contains a relatively low number of bacteria with less diversity [73,74,75]. Characterization of gut microbiota has found that the dominant phyla in healthy humans are the *Firmicutes* and *Bacteroidetes*, while other species such as *Proteobacteria*, *Actino-bacteria*, *Fusobacteria*, and *Verrucomicrobia* are present in lesser amounts [76]. In contrast, the major phyla constituting the gastric microbiota are *Proteobacteria* and *Firmicutes*, along with *Bacteroidetes*, *Actino-bacteria*, and *Fusobacteria* [72]. However, one characteristic of commensal bacteria in humans is their instability and dynamic nature. The composition of gastric microbiota is subject to change during aging, diet ingests, drug use (especially proton-pump inhibitors), *H. pylori* infection, and alterations of local immunity [77,78]. The pathobiont bacteria, *H. pylori*, once acquired, can live symbiotically for a varied period in gastric mucosa without pathogenic effects [79]. Once the balance between host defense mechanisms and *H. pylori* invasions is disrupted, *H. pylori* can become the dominant bacterium in the gastric mucosa, causing chronic gastritis and potentially leading to carcinogenesis [80].

ILCs have long been recognized to have substantial crosstalk with the gut microbiota. The interaction between ILCs and the gut microbiota can maintain mucosal homeostasis on the one hand and induce downstream immune reactions on the other. However, studies on ILCs and their relationship with gastric microbiota are comparatively limited. The general impression that microbiota and ILCs have complicated and bidirectional interactions, which has been confirmed in the setting of the intestinal tract, might also be applied in the scenario of the gastric mucosa. ILCs can limit the overgrowth of pathogenic bacteria by producing cytokines and anti-microbial peptides, enhancing mucosa integrity, and activating the downstream adaptive immune system [8]. In turn, the development, activation, and function of gastric ILCs can be induced and modulated by the gastric microbiota. One example is that the induction of cytotoxicity and augmentation of IFN-γ production by NK cells is observed during *H. pylori* infection [81,82,83]. ILC2s, as the dominant component of gastric ILCs, are also believed to rely on their development on gastric microbiota in an IL-7R-dependent manner [46]. In a germ-free mouse model, a sharp decrease of ILC2s in gastric mucosa is observed, whereas intestinal or bronchial ILC2s are unaffected by the absence of commensal bacteria [84,85]. A significantly high expression of IL-7R on gastric ILC2s compared to other tissues indicates that they rely on the activation signal input via IL-7, which is induced in the stomach by commensal bacteria [46]. Meanwhile, commensal bacteria can induce the secretion of IL-7 and IL-33 in the gastric mucosa and, in turn, trigger the proliferation and activation of ILC2s, enhancing their defense against pathogens such as *H. pylori* [85]. Among gastric microbiota, a *Bacteroidales* species has been shown to be a candidate bacterium for ILC2 activation [46].

In conclusion, priming by commensal bacteria, on the one hand, ensures the proper development and activation of ILCs. In turn, primed ILCs can augment mucosal integrity and exert their anti-pathogen effects. The gastric ILCs, especially ILC2s, are vital in maintaining the homeostasis of gastric microbiota, limiting the overgrowth of pathogenic microbes, and potentially reducing the risk of oncogenesis.

## 4. ILCs and *Helicobacter pylori* Infection

*H. pylori* has been tightly linked to gastric cancer since its official categorization as a human group I carcinogen over a decade ago [86]. Prolonged *H. pylori*-induced gastritis leads to gastric cancer following Correa’s cascade [87], but eradication therapy has been shown to reduce the incidence of, or prevent, gastric cancer development [88]. Unlike other gastrointestinal pathogens, *H. pylori* infection generally occurs in childhood through oral transmission and shows an immensely high degree of familial aggregation [89]. If not treated, it can persist for life [89]. During *H. pylori*’s pathobiont persistence in the harshly acidic gastric environment, it reshapes the gastric ecology by damaging the mucosa via its virulence factors, which leads to modulation of host inflammatory response and alteration of local hormone release patterns, resulting in an abnormal status with sensitivity and fragility, including microbiota [90]. In gastric inflammation and oncogenesis caused by *H. pylori*, various immune reactions have been activated to propel the normal mucosa toward intestinal metaplasia, dysplasia, and even carcinoma [91], represented by WNT–β-catenin signaling, NF-κB, and TLR4/5/9 [92,93,94,95]. Among the innate immune responses to *H. pylori* infection, ILCs are pivotal in skewing different axis of immune responses and activation of downstream adaptive immune reactions.

It is well established that specific protein virulence factors, lipopolysaccharide, and Hop proteins present in *H. pylori* play crucial roles in the pathogenesis of *H. pylori* infection [81,96]. These constituents facilitate the colonization of *H. pylori* in the gastric mucosa and interact with various host cells and molecules, leading to complex immune responses through different pathogenic signaling pathways [81,96]. Among the infiltrating immune cells in gastric cancer patients, natural killer (NK) cells are one of the most representative and functionally significant cells. NK cells form the first immune barrier against *H. pylori* invasion in the human gastrointestinal mucosa. Upon *H. pylori* infection, NK cells rapidly proliferate and activate, augmenting their cytotoxic effects and secreting interferon-gamma (IFN-γ) to trigger a solid local inflammation. The reorganization of HpaA via the Toll-like receptor 2/1 (TLR2/1) complex with MyD88 and p38 MAPK further enhances the cytotoxic effects of NK cells against *H. pylori*. Importantly, this entire process is synergistically enhanced by interleukin-12 (IL-12) produced from macrophages and dendritic cells in the *H. pylori*-infected gastric mucosa [81,97,98,99].

Despite the sophisticated and vigorous protective immune response elicited by *H. pylori* infection, some individuals still face a poor prognosis of developing gastric cancer [100,101]. As one of the major culprits in immune system breakdown and malfunction, *H. pylori* components can be double-edged swords in terms of their effect on the immune system. Although they can stimulate the host’s immune response, the persistent Th1 immune response aroused by NK cells may contrarily contribute to the development and progression of gastric oncogenesis [102]. Several studies have also demonstrated that both specific protein virulence factors and lipopolysaccharides have negative immunomodulatory effects. They can directly downregulate the natural cytotoxicity by diminishing the ability of NK cells to produce IFN-γ and IL-2, accompanied by lacking CD3^−^CD56^+^CD25^+^ NK cells [103], while retaining IL-10 production, represented by CD8^−^CD16^−^CD56^bright^ NK cells [104]. Additionally, perforin production by NK cells is downregulated [105]. Interestingly, *H. pylori* can even directly promote the growth of gastric cancer through the LPS–TLR4 pathway, and vice versa, the neutralization of TLR4 can suspend this proliferative activity [106].

In addition, HopQ, an outer-membrane adhesion protein of *H. pylori*, has been shown to bind to carcinoembryonic antigen-related cell adhesion molecules 1 (CEACAM1), an inhibitory receptor expressed by activated NK cells, which can directly impair NK cell function [107]. Meanwhile, Hp(2-20), a cecropin-like *H. pylori* peptide, can attract and activate monocytes to produce oxygen radicals, which can further attenuate NK cell-anti-tumor cytotoxicity and promote apoptosis [108]. This phenomenon has also been observed with *H. pylori* lysate and the synthetic bacterial lipoprotein FSL-1, which are thought to be associated with tumor-derived TGF-β and the transcription factor, GATA-3 [99].

ILC2s, the predominant subset of helper-like ILCs in gastric mucosa [46,47], have also been illustrated as critical in controlling chronic *H. pylori* infection. *H. pylori* infection clearly skews the immune response toward type 2 immunity mediated by ILC2s and GATA-3 compared to a decreased Th1-axis immune response [47]. The expression of GATA-3, a signature marker of ILC2s, is significantly upregulated after *H. pylori* infection and leads to a decrease in Connexin43 (Cx43), a major constituent of gap junctions in the normal gastric mucosa that contributes to the development of gastric cancer [109]. However, there is also evidence showing that, during *H. pylori* infection, gastric ILC2s can be activated by IL-7 and secrete IL-5, subsequently activating B cells into IgA-producing plasma cells rapidly, showing vigorously protective capability for gastric mucosa [46]. This controversy between the two distinct points is most likely caused by the different immune responses induced by acute and chronic infections. IL-33, as a stomach alarmin, is increased with high fold immediately after gastric insult and infection to effectively activate ILC2s and T cells to initiate type 2 immune response, which may be beneficial by preventing unchecked Th1/Th17 inflammation and showing protective effects. However, as the infection course prolongs, the concentration of IL-33 reaches a peak and subsequently decreases, gradually showing a tendency to promote tumor through producing M2 macrophage polarization and increased STAT3 activation [110]. Consistent with that, the expansion of stomach ILC2s of *H. pylori*-infected mouse models reaches a peak in two weeks, indicating that the course of infection might alter local immune responses carried out by ILC2s [46].

To sum up, during pathobiont persistence, *H. pylori* damages mucosa via its virulence factors, modulating host inflammatory response in diverted ways. *H. pylori* components are double-edged swords that can stimulate host immune responses or act as negative immunomodulators to NK cells, one of the major cytotoxic infiltrating functional immune cells. Among the helper-like innate immune responses to *H. pylori*, ILC2s also play a dual role in limiting *H. pylori* infection and oncogenesis, most likely in the infection course-dependent manner.

## 5. Roles of ILC Subsets in Gastric Oncogenesis

### 5.1. Group 1 ILCs: NK Cells and ILC1s

The expression of CXCR6 and integrin α4β7 allows ILC precursors to have a tendency to migrate and reside in the gastrointestinal tract [12]. Moreover, in the presence of retinoic acid at the early stage of growth, integrin α4β7 and CCR9 show increased expression. In contrast, CCR7 shows reduced expression, further aggravating the migrating and residing propensity, especially for ILC1s and ILC3s [111]. Meanwhile, the cytotoxicity property of NK cells and the typical capacity of NK cells and ILC1s to produce IFN-γ and TNF enables them to exert strong immune reactions against viruses and tumors [112]. All the above provides a fundamental basis for Group 1 ILCs to participate in gastric oncogenesis.

The immunosurveillance function and direct cytotoxic effect against cancer cells of NK cells are well-recognized in various types of malignancies with well-explained mechanisms, as illustrated above. Specifically, in the setting of gastric cancers, several studies have demonstrated that Group 1 ILCs, particularly NK cells, have a role in the anti-tumor immune response. A significant accumulation of Group 1 ILCs within the malignant lesions compared with surrounding normal tissues indicates the immensely intense immune response in the neoplastic area mediated by these cells [113]. T-bet+ cells and NK cells infiltration into tumorous tissues has a positive relationship with improved prognosis in patients with gastric cancer, including disease-free survival and overall survival [114]. Nonetheless, the role of Group 1 innate lymphoid cells in the progression of gastric cancer is not always inhibitory.

As aforementioned above, NK cells tell “non-self” from “self” via their modulating their activating and inhibitory receptors, marked by an elevated expression of DNAX accessory molecule-1 (DNAM-1) and decreased expression of MHC Class I. However, cancer cells are smart enough to deceive this mechanism by modulating the expression of carcinogenic ligands and NK cell receptors. Consequently, in gastric cancer patients, the phenotype of NK cells is often suppressive, characterized by downregulated activating receptors such as NKG2D and upregulated inhibitory receptors [115,116,117]. The highly expressed 9-27 gene in gastric tumor tissue and cancer cells induced by IFN-γ can reduce the susceptibility of tumor cells to NK cells by delivering negative signals, as 9-27 is a component of several membrane proteins such as CD81 [118]. Overexpression of 9-27 can also enhance the migration and invasion of gastric cancer cells, leading to a stronger propensity for malignancy [118].

ILC1s are a unique subset of ILCs with potent type 2 interferon-production capability and none or limited cytotoxic potential, which sets them apart from NK cells [23]. However, the precise roles of ILC1s in gastric cancer are still unclear. The IFN-γ produced by ILC1s plays a critical role in inducing Th1-axis immune response, leading to downstream activation of cytotoxic T lymphocytes, NK cells, macrophages, and other phagocytes and ultimately contributing to anti-tumor immune responses [119]. Additionally, IFN-γ can augment the expression of MHC class I on cancer cells, which favors direct cytotoxicity mediated by cytotoxic T cells [120]. Furthermore, IFN-γ exhibits strong direct anti-tumor effects by inhibiting angiogenesis and cancer cell proliferation and inducing apoptosis [121]. However, IFN-γ may also promote tumorigenesis via their contributions to chronic inflammation [121]. The relationship between excessive exposure to IFN-γ and tumor growth has been observed in several studies [122,123,124]. IFN-γ may also induce the expression of multiple immune checkpoint ligands, including programmed death-ligand 1 (PD-L1) and PD-L2 on tumor cells, and, therefore, attenuates the anti-tumor response mediated by T cells and NK cells [125,126]. Nevertheless, these indirect pieces of evidence may not fully explain the anti-tumor immune response mediated by ILC1s in gastric cancer. Therefore, future studies may focus more on investigating the roles of ILC1s and their respective signature cytokines in innate immune responses to gastric cancer.

The commonly accepted concept is that NK cells and ILC1s mediate the anti-tumor effect via their direct cytotoxicity and type 2 interferon production. However, in the setting of gastric tumors, Group 1 ILCs may also be altered to favor oncogenesis via the change of activating and inhibitory-receptor expression. In the treatment of gastric cancers, direct adoptive transfer of NK cells or enhancement of NK cell and ILC1 anti-tumor function by targeting the inhibitory receptors of NK cells and ILC1s such as NKG2A may serve as a potential therapeutic strategy, which has been under investigation in colorectal cancers [39].

### 5.2. Group 2 ILCs

Innate lymphoid cells type 2 (ILC2s) are the innate counterparts of type 2 helper T (Th2) cells and their constitutive expression of receptor subunit ST2 is crucial for recognizing and binding with cytokines such as IL-25 and IL-33, which are produced by epithelial cells. This binding initiates downstream signaling and results in the abundant production of type 2 cytokines, including IL-5, IL-9, IL-13, and amphiregulin [127]. The transcription factors GATA3 and RORα are key effector elements in this process [127]. Although only a few ILC2s are found in the peripheral blood due to their tissue-resident properties [128], their frequency is significantly increased in the peripheral blood of gastric cancer patients, indicating their potential involvement in gastric oncogenesis [129].

Unlike Group 1 innate lymphoid cells, the majority of ILC2s are protumorigenic and rely on creating an immunosuppressive environment. This correlation has been shown in a mouse model with gastritis and gastric cancer, where isthmus stem cells exhibit a proliferative tendency with the expansion of CXCL12+ endothelial cells and CXCR4+ ILC2s, in which the inflammatory CXCL12/CXCR4 chemokines has been thought as an important signaling molecule in gastritis and gastric cancer [130]. In turn, this phenomenon can be effectively interrupted by genetic deletion of CXCL12, pharmacological inhibition of CXCR4, and ablation of ILC2s. Meanwhile, disrupting CXCL12/CXCR4-mediated signaling pathways also contributes to inhibiting further metastasis of gastric cancer at a later stage [130]. Notably, the increased ILC2s in peripheral blood from gastric cancer patients are evidently related to the Th1/Th2 imbalance and the upregulation of MDSCs and M2 phenotype macrophages, which both play a noted immunosuppressive role in the tumor microenvironment [129,131,132]. In gastric tissues from patients with spasmolytic polypeptide expressing metaplasia (SPEM) under the background of severe gastric injury, a significant increase in the number of ILC2s and the elevated ILC2-induced type 2 inflammation coordinates the metaplastic response to gastric injury [133]. Amphiregulin, a major production by ILC2s, which possesses the ability to promote tissue invasion and metastasis of epidermal growth factor receptor-expressing tumors [134], has been shown to favor tumorigenesis and progression in a series of cancer models, including gastric cancer [135]. In addition, in eosinophilic gastritis, which belongs to eosinophilic gastrointestinal disorders characterized by eosinophil, mast cell, and B cell infiltration-associated inflammation and characterized as precancerous lesions, ILC2s contribute to the pathogenesis via their production of IL-5 and IL-13 induced by IL-33 and TSLP [136,137,138,139,140].

Recent studies have shown that targeting ILC2s can have therapeutic potential in preventing gastric inflammation and cancer development, and even reversing the pathological changes associated with metaplasia after disease onset. Attempts have been made in mouse models through genetic ablation of ILCs or antibody-mediated blockade of IL-13 or IL-25, which can reduce the growth of gastric cancer [141]. More specifically, ILC2 depletion via treatment with CD90.2 antibodies has also been shown to directly protect the stomach from SPEM development [142]. Administration of exogenous androgen (5α-dihydrotestosterone, DHT) into male mice with removal of endogenous glucocorticoids and male sex hormone by adrenalectomy and castration prevents SPEM development and reverse metaplastic changes associated with gastric injury, potently via the suppression of IL-13 and CSF2 expression from ILC2s [142]. The mechanism that ILC2 may contribute to gastric inflammation, SPEM development, and gastric oncogenesis in an androgen and glucocorticoid-dependent manner explains the protumorigenic function of ILC2s in a novel way.

In summary, ILC2s play a critical role in gastric oncogenesis through their protumorigenic effects, including the production of type 2 cytokines, promotion of chronic inflammation and tumor development, the polarization of macrophages towards M2 phenotype, and interaction with other immune cells such as MDSCs and M2 phenotype macrophages. A direct relationship between ILC2 ablation and reduced risk of gastric tumorigenesis has been observed. Hence, targeting ILC2s to inhibit their function may have great potential in finding novel treatments for gastric cancers.

### 5.3. Group 3 ILCs

Compared to the other two types of ILCs, ILC3s are predominantly found in the intestine and are concentrated in cryptopatches, lymphoid follicles, and throughout the lamina propria [143,144], where they have an inextricably reciprocal relationship with the symbiotic enteric bacteria [8]. Except for tissue residency and homing mediated by CXCR6 and integrin α4β7 as mentioned above, lymphatic networks also provide another possibility of ILC3 for migrating between the mesenteric lymph node and the gastrointestinal tract [127]. The pivotal function of ILCs for the extracellular microbiota is to secrete IL-22 and IL-17 in response to IL-23 and IL-1β, similar to Th17 for type 3 immunity, through RORγt and ID2-dependent manner. And several studies have reported that it also can exert immunomodulatory by secreting additional cytokines such as IL-26, GM-CSF, and TNF-α [16] or by transdifferentiating from NCR+ ILC3s into ILC1s upon IL-1β and IL-12 [17], or by expressing MHIC-II molecules to present commensal bacteria antigens to CD4+ T cells [145]. Although ILC3s abnormalities have been shown to be strongly associated with sustained inflammatory states and contribute to inflammation-related tumorigenesis, mainstream studies are mainly focused on gut cancer and a few sporadic studies about hepatic cell carcinoma and pancreatic cancer [146,147]. Intriguingly, the role of ILC3s in those studies was surprisingly consistent regarding tumor-promoting and metastasis activities. Regarding the mechanism of gut cancer, it is now thought to be associated with cytokines such as IL-17 and IL-22 that can mediate pro-tumorigenic STAT3 activation [148] IL-23/IL-23R^+^ILC3s/IL-17 cascade, ILC3s/IL-22 shuttle and the transdifferentiation from ILC3s into regulatory ILCs upon TGF-β stimulation [149].

However, the role of ILC3s in gastric cancer is not well understood. Studies have shown that the levels of ILC3s and IL-22 in patients with gastritis, precancerous lesions, and gastric cancer are significantly higher than those in healthy populations, suggesting that ILC3s may play a role in gastric oncogenesis and have important immune functions [150]. Nevertheless, further high-quality studies are needed to deeply reveal and understand the relationship between ILC3s and gastric oncogenesis, especially regarding molecular mechanisms.

## 6. Concluding Remarks

**Figure 1:**
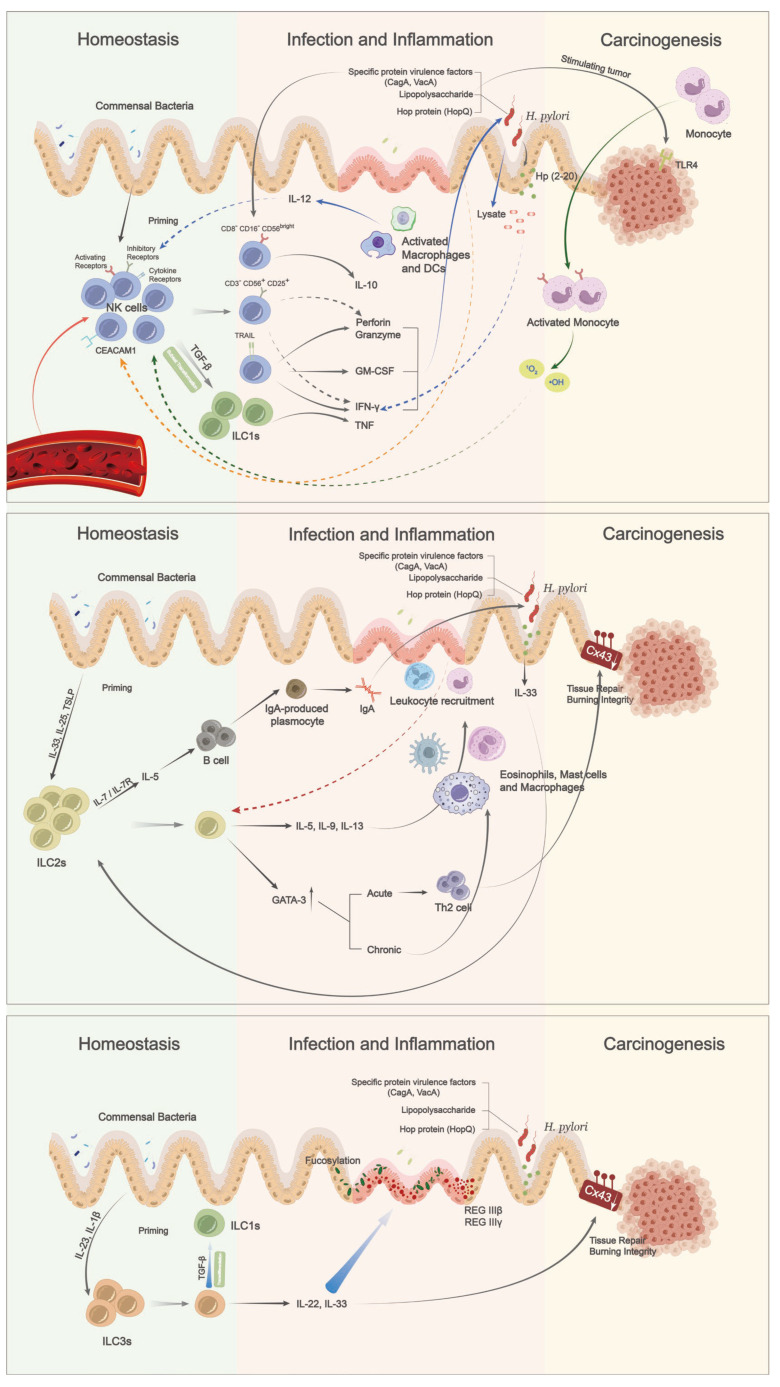
ILCs in regulating gastric mucosal homeostasis and their roles in gastric cancer. Group 1 ILCs consists of cytotoxic NK cells and ILC1s. NK cells rely on mucosal commensal bacteria for their priming and allow for future function. NK cells limit *H. pylori* infection via direct cytotoxicity and the production of IFN-γ. In turn, *H. pylori* may also produce virulence factors and specific peptides to attenuate NK cell anti-tumor effect. Meanwhile, activated macrophages, DCs, and monocytes are also getting involved in this process. ILC2s may have dual roles in both controlling over *H. pylori* infection and gastric oncogenesis via multiple mechanisms, with the help of eosinophils, mast cells, and macrophages. As the number of ILC3s is limited in gastric mucosa, their actual function remains uncertain. Contrastly, mainstream studies are mainly focused on the perspective of gut, parts are selected to provide the potential reference, labeled by thick arrows with blue gradient.

ILCs have emerged as key players in regulating immune responses and maintaining tissue homeostasis in various organs, including the intestinal tract and stomach. The expression of tissue-homing receptors indicates their tissue-residency property, making them mainly located in mucosal tissues to form the first-line defense system. Their innate immunity nature, characterized by multiple activating, inhibitory, and cytokine receptors without the need for specific antigen receptors, enables a rapid and robust innate immune response and strong immunosurveillance potential against infection and cancer cells (Figure 1).

Mucosal ILCs have extensive crosstalk with exogenous pathogens, and their relationship with local commensal bacteria plays a crucial role in their activation and functionality, known as the “priming” process. Mature and properly activated gastric ILCs limit pathogenic invasion and contribute to local tissue repair, particularly in controlling *H. pylori* infection. Accumulating evidence has shown that ILCs may contribute to immune responses to *H. pylori* infection via direct cytotoxicity, induce pro-inflammatory immune responses, and promote downstream adaptive immune responses. Their role in limiting *H. pylori* infection serves as a critical protective mechanism to reduce the risk of gastric cancer.

The roles of ILCs in different types of cancers have been comprehensively studied, and it is suggested that they may have dual roles in tumorigenesis (Table 1). Even classical anti-tumor immune responders, such as NK cells, are found to mediate tumor growth in certain conditions potentially. In the setting of gastric cancers, ILC2s are also found to be both pro-tumorigenic and anti-tumorigenic according to different studies from different aspects. However, the current understanding of the exact role of ILCs in gastric cancer is still limited.

Most studies on immunotherapies to cancers have been focusing on cytotoxic T cells, antigen-presenting cells, and other subsets of regulatory cells. Remarkable achievements have been made owing to these findings and clinical translations. However, the limitations and adverse events of these therapies have always been a challenge in clinical application. Fully understanding the immune landscape of different cancers and deciphering the roles of each immune subset may open up novel possibilities to improve treatments for gastric cancers.

## Figures and Tables

**Table 1:** Roles of ILCs in Promoting or Inhibiting Gastric Oncogenesis *.

Cell Type	Function	
	Anti-Tumor	Pro-Tumor
NK cell	Accumulation of NK cells and ILC1s in gastric cancer tissue [113] NK cell infiltration positively related to improved prognosis [114]	Downregulation of activating receptors and upregulation of inhibitory receptors [115,116,117] 9-27 gene expression in gastric cancer cells reduces NK cell cytotoxicity [118]
ILC1	Accumulation NK cells and ILC1s in gastric cancer tissue [113]	N.A.
ILC2	N.A.	CXCR4+ ILC2s correlate with isthmus stem cell proliferation and may contribute to tumor growth [129] Inhibition of CXCR4 may inhibit tumor growth [129] Increased peripheral ILC2s in gastric cancer patients correlate with the upregulation of MDSCs and M2 macrophages [131,132,133] ILC2s and Th2 immune responses correlate with SPEM [134] Amphiregulin produced by ILC2 promote EGFR^+^ tumor growth [135] ILC2s favor the precancerous lesion eosinophilic gastritis via their production of IL-5 and IL-13 induced by IL-33 and TSLP [137,138,139,140,141] Ablation of ILC2 or IL-13/-25 blockade reduces gastric cancer growth [142,143] ILC2 contributes to gastric inflammation and SPEM development in a glucocorticoid and androgen-dependent manner [143]
ILC3	N.A.	Elevated ILC3 and IL-22 levels in gastritis and gastric cancer patients (specific roles uncertain) [150]

N.A., not applicable. * Indirect evidence is not listed in this table.

## Data Availability

Not applicable.
